# Parallel NGO Networks for HIV Control: Risks and Opportunities for NGO Contracting

**DOI:** 10.5539/gjhs.v5n2p171

**Published:** 2012-12-27

**Authors:** Shehla Zaidi, Xaher Gul, Noureen Nishtar

**Affiliations:** 1 Department of Community Health Sciences Sciences, The Aga Khan University, Karachi, Pakistan; 2 Women and Child Health Division, The Aga Khan University, Karachi, Pakistan

**Keywords:** HIV, Pakistan, NGO Contracting, NGO parallel networks, Public Private Partnership

## Abstract

Policy measures for preventive and promotive services are increasingly reliant on contracting of NGOs. Contracting is a neo-liberal response relying on open market competition for service delivery tenders. In contracting of health services a common assumption is a monolithic NGO market. A case study of HIV control in Pakistan shows that in reality the NGO market comprises of parallel NGO networks having widely different service packages, approaches and agendas. These parallel networks had evolved over time due to vertical policy agendas. Contracting of NGOs for provision of HIV services was faced with uneven capacities and turf rivalries across both NGO networks. At the same time contracting helped NGO providers belonging to different clusters to move towards standardized service delivery for HIV prevention. Market based measures such as contracting need to be accompanied with wider policy and system measures that overcome silos in NGO working by facilitating a common construct on the health issue, cohesive priorities and integrated working.

## 1. Introduction

There has been an increased emphasis on contracting of Non-Governmental Organizations (NGOs) for service delivery in recent years (Perrot, 2006). Contracting is a neo-liberal based measure commonly involving open market competition for specified, time bound and result oriented services (Taylor, Preker, & Harding, 2003). National responses for HIV control often rely on contracting NGOs for reaching disadvantaged groups at high risk for HIV/AIDS such as sex workers, injectable drug users, etc. In developing countries, large donor funded initiatives such as the World Bank Multi-country AIDS Program (MAP), the US President's Emergency Plan for AIDS Relief (PEPFAR) and the Global Fund to Fight AIDS, Tuberculosis and Malaria (GFATM) have provided the drive for policy measures to address HIV. Policy measures have often assumed a homogenous NGO sector and an even market response to contracting. However in actuality, non-state actors may well comprise of distinct NGO groups working in parallel. Market dynamics of NGOs in response to contracting have been less well explored despite the centrality of NGOs to contracting. We take up a case study of Pakistan and comment on the parallel NGO networks in existence for HIV response, their distinctive approaches, and the risks and opportunities for market-based approaches such as contracting.

## 2. The Pakistan HIV Case

Pakistan is a country with less than 1 per cent prevalence of HIV but at high risk of spread (World Health Organization, 2009). Infection is negligible in the general population and is mainly confined to high risk groups with 5-12 per cent HIV prevalence in Injection Drug Users (IDUs) and male se workers (MSWs), followed by less than five per cent prevalence amongst female sex workers (FSWs), long distance truckers and prisoners (Ministry of Health, 2005). Historically, Pakistan has treated HIV control as a separate policy agenda, managed by the National AIDS Control Program (NACP). HIV has received low policy support due to its taboo nature and this together with vertical management, has resulted in little integration of HIV into public sector health facilities (Zaidi, Mayhew, Cleland, & Green, 2012).

## 3. Hybrid NGOs and NGO Networks

There were as many as 350 local NGOs registered to be involved in HIV prevention with UNAIDS (2004). However mapping studies and experts estimated active NGOs to be much fewer (Greenstar Social Marketing, 2002). Of the active NGOs, 6-10 NGOs came predominantly from a Reproductive Health (RH) background with some experience in HIV. Another 20-30 were known mainly for HIV centered work and comprised a hybrid group of NGOs. The rest were little known and mostly inactive (Interact Worldwide, 2004). The HIV and RH NGO communities had thus far led largely separate and compartmentalized work existence.

### 3.1 Evolutionary & Organizational Features

HIV NGOs were mostly formed during the mid-1990s and comprised of small NGOs from diverse backgrounds. In the absence of a clear ideology or strategic direction, these were best known for their identification with the HIV issue or identification with a particular high-risk group. These worked in discrete localities with an identified risk group using indigenous sources of funding. Organizationally, the majority of the HIV NGOs were under-developed; ranging from one person led outfits to small and at best medium scale organizations having a staff of 5-10 persons (Interact Worldwide, 2004). Many lacked adequate office space, written mission statements, proper official documentation and program monitoring systems. The main strength of HIV NGOs over and above other organizations were linkages and familiarity with high risk groups who because of their quasi-legal status and taboo practices were provided scant cover by both public and non-state sectors.

The RH NGOs had evolved during the 1980s and 1990s as Reproductive Health incrementally received policy recognition and replaced older agendas of population control and primary health care (PHC) implementation. These included some of the most developed and dominant NGOs in the public health sector and drew ideological identification mainly from the ICPD 1994 with a focus on women's health and integrated service provision. RH NGOs comprised of large to medium sized organizations (some even having a countrywide infrastructure), were headed by many of the local, leading public health experts and were usually internationally funded. RH NGOs were experienced in program management, had well developed work organograms, offices and services infrastructure. Of the RH NGOs, five provided HIV prevention services through counseling and condom promotion and another two were involved in sexual health advocacy, which included HIV, amongst other issues.

### 3.2 Clientele and Nature of Work

Both types of organizations had largely separate clientele and this shaped the type of services provided. The HIV NGOs were involved with a specialized clientele at higher risk of HIV infection that comprised of FSWs, IDUs, transvestites, distance truckers, cross-border traders and prisoners. Activities in the general population were few and targeted the youth and community stakeholders such as police personnel and religious leaders, rather than married women. Activities mainly related to awareness raising on World AIDS Day walks, awareness seminars, organization of medical camps for HIV awareness or small scaled screening projects in risk groups or provision of charitable services amongst quasi-legal groups and people living with AIDS. (UNAIDS, 2004; Greenstar Social Marketing, 2002) Involvement with service delivery was rare and such projects when undertaken remained confined to HIV screening and testing, underwritten by an emphasis on infection control rather than behavioral change. Although they had links with at-risk groups, coverage was limited; estimated at five per cent of the total risk group population (World Bank, 2001). The often sporadic and short tenured nature of their work blunted their in-depth experience with at risk groups.

The RH NGOs, in contrast, were positioned towards married females in the general population. These NGOs faced little demand from their predominantly married female clients for sexually transmitted illnesses (STI) or HIV prevention services. HIV services provided by RH NGOs mostly consisted of awareness provision and at most stretched to screening and counseling rather than the full range of behavior change communication, STI treatment, care, support and empowerment services recommended by UN as part of HIV prevention (UNAIDS, 2005). At least two RH NGOs had moved into condom distribution to males in the general population and long distance truck drivers for HIV and STI prevention, however, male targeting by the rest was weak. Three other NGOs, known for their research capacity, had been commissioned by development partners for occasional small-scale HIV related research. Generally, there was weak involvement with quasi-legal groups most at risk for HIV/AIDS.

### 3.3 Networks of Interaction

Separate NGO networks existed for HIV NGOs and RH NGOs. The HIV focused NGOs participated in the HIV NGO consortia initiated by UNAIDS in 1999 which served as a platform for informal interaction between NGOs and provincial AIDS Control Programs. This was a registered and active forum where HIV focused NGOs exchanged information, learnt about new programmatic initiatives and at times, formed collaborative linkages. On a parallel footing, RH NGOs participated in a number of overlapping networks including the Pakistan Reproductive Health Network, women's rights networks and the Population Association of Pakistan. These have ideologically underpinnings of women's empowerment and right to sexual health as provided for in the International Conference on Population and Development (ICPD) 1994 and the Fourth World Conference on Women (FWCW) in Beijing 1995.

There were also essential differences in stakeholder interaction with government and development partners. For HIV focused NGOs, the NACP provided the major government interface while UNAIDS constituted the major international resource body. Dealings of HIV NGOs followed the vertical orientation of the NACP and had little involvement with mainstream public health infrastructure where HIV has yet to be integrated with other services. Conversely, the RH NGOs being involved with mainstream RH and PHC services liaised with a number of different entities including the Ministry of Population Welfare, Ministry of Health, WHO, UNICEF and UNFPA. They had at best occasional interaction with the separate policy network of the HIV actors.

## 4. Contracting for HIV Prevention

In 2003 an aggressive national HIV control response was initiated with international donor support and relied extensively on NGO involvement for HIV control. Promising local NGOs were contracted to provide HIV prevention services in high-risk groups such as IDUs, MSWs, FSWs, jail inmates, and borderline groups such as long distance truckers and youth.

### 4.1 Commonality in Services and Stakeholder Interaction

Nearly all NGOs active in HIV applied for contracts and included both RH and HIV NGO clusters. Contractual service packages ranged from tightly standardized packages detailing services and performance deliverables supported by World Bank and DFID to less standardized packages supported by European Commission and USAID. Under contracts, the HIV NGOs shifted from mere awareness generation activities to service delivery while RH NGOs already involved with sexual health counseling expanded into full range of HIV prevention services.

Both sets of NGOs also started interacting with a common set of policy stakeholders involving the AIDS Control Program, international donors investing in HIV and International NGOs managing contracting. However, as HIV remained a vertical response, there was little interaction with other vertical programs dealing with maternal and reproductive health. HIV programs continued to be implemented through NGO outlets while reproductive health continued separately through government mainstream facilities and community health workers.

### 4.2 Capacity Snags and Opportunities

Both NGO communities were under-equipped to meet the requirements of service delivery contracts for HIV. RH NGOs were able to write and negotiate sophisticated bids for HIV contracts and provided seasoned program management, monitoring and documentation but lacked links and experience with risk groups. Conversely, HIV NGOs were familiar with risk groups but lacked capacity to work at scale. Capacity building in varying measure took place alongside contracts with both sets of NGOs gaining experience in programming service delivery for risk groups. Contractual experience also encouraged NGOs to take on further HIV service delivery contracts. However, capacity building through on ground experience was a slow process and performance except for few contracts remained lower than stipulated targets (Zaidi et al., 2011).

### 4.3 Partnerships and Rivalry

Although both NGO clusters had complimentary capacities, there were fewer than expected partnerships between HIV and RH NGOs. NGOs preferred to form partnerships with like-minded groups. Even in instances where contractual partnerships were undertaken between larger RH NGOs and smaller HIV NGOs, these did not continue over in subsequent contracts after some level of capacity was built in both NGO clusters. Both HIV and RH NGOs continued to associate in separate NGO networks. Network boundaries in fact sharpened in the wake of donor funded HIV contracts, driven by underlying suspicions of fund driven participation for HIV contracts. Some NGOs raised concern on lack of over-riding focus on service delivery and lack of effort put into developing common ideological ground and commonality of approach within respective NGO communities. There were also fears of sustainability whether NGOs would continue to be engaged with HIV prevention and control after funding for contracts ended.

## 5. Is Contracting Affected by Parallel NGO Clusters?

So far the compartmentalization debate has typically centered on government vertical programs (Richey, 2003) and its purview has not extended to non-state actors. The separation of HIV control globally from the larger Reproductive Health (RH) agenda and resulting emergence of parallel planning, implementation and monitoring systems has been well documented in published literature (Biesma et al., 2009; Liu, Hotchkiss, & Bose, 2007). What has been less well recognized is that vertical policy responses for HIV and RH at both global and national levels have also been instrumental in shaping parallel communities of non-state actors.

In Pakistan, the HIV and RH NGO communities were historically different in terms of ideology, organizational features, programmatic goals, clientele and stakeholder linkages. These networks tended to work in parallel and derived their distinctiveness from vertically driven policy responses to HIV and RH in Pakistan. We found that both their distinctive features and lack of connects in working conditioned their response to HIV contracting.

Contracting for HIV prevention was faced with uneven capacity of both sets of NGOs involving weak contract and project management skills in the HIV focused NGOs and sub-optimal contextual knowledge of risk groups amongst the larger RH NGOs. Hence none of the winning bidders was an ideal candidate for implementing HIV contracts. In the long run contracting became a means to overcome capacity gaps by providing grounded experience through contracts. Market stimulation through contracts also instigated both communities of NGOs into providing fairly uniform and standardized HIV prevention services that were underwritten by tight performance based contracts. Contracting brought NGO groups together for service delivery projects, however competition for service contracts in the absence of larger policy harmonization efforts engendered friction and rivalry, with preference for bidding partnerships with like-minded groups.

Much has been written in recent years about contracting of NGOs for health service provision in developing countries. However the focus of research has been on quantitative results of contracting, mainly service coverage expansion (Loevinsohn & Harding, 2005). There still remain unanswered questions about dynamics of the NGO market around contracting which can be an important influence the number and strength of bids and quality of implementation. Contracting in developing countries, particularly in states where it is extensively practiced, has often been dominated by international NGOs as seen in Afghanistan, Haiti, and Cambodia (Ridde, 2005; Eichler, 2001; Soeters, 2003). It has been noted that there is less known about capacity of local NGOs and their motivations to contract (Palmer, 2006). At the same time, the policy context within which NGOs evolve and contracting is implemented is an important variable that remains under-explored (Zaidi, 2012; Liu, 2008). This paper addresses knowledge gaps in two main areas. First, it adds to qualitative literature on contracting specifically exploring the dynamics of the NPO market around contracting. Contracting was adversely affected by fragmented NGO communities working in silos, even though contracting in the long run helped overcome compartmentalized working to an extent. Second, in this paper we related the market dynamics with the policy context within which NGOs evolve emphasizing the need for wider policy efforts to reduce compartmentalized working within NGOs.

Based on the above we recommend, that market based efforts, such as contracting, need to be accompanied by wider policy and systems efforts for furthering productive interaction between parallel NGO communities. Such efforts need to be aimed across a spectrum of NGO stakeholders at developing a common construct on the issue and a cohesive common understanding of policy responses. Moreover policy measures need to operationally encourage integrated working across similar health programs such as HIV and RH (Lusti-Narasimhan, Say, & Mbizvo, 2010; Askew & Berer, 2003). Measures involving shifts in policy paradigms may however take longer to bed down than more operational responses such as contracting.

## 6. Conclusion

Contracting for HIV control can overcome compartmentalization of NGOs by bringing providers towards more standardized service packages but is also negatively affected by market deficiencies and sharpened rivalries as a result of compartmentalization. Wider policy and systems measures are needed in addition to contracting to overcome parallel provider networks.
